# Adipocyte STAT5 deficiency does not affect blood glucose homeostasis in obese mice

**DOI:** 10.1371/journal.pone.0260501

**Published:** 2021-11-24

**Authors:** Marianna Beghini, Theresia Wagner, Andreea Corina Luca, Matthäus Metz, Doris Kaltenecker, Katrin Spirk, Martina Theresa Hackl, Johannes Haybaeck, Richard Moriggl, Alexandra Kautzky-Willer, Thomas Scherer, Clemens Fürnsinn

**Affiliations:** 1 Division of Endocrinology & Metabolism, Department of Medicine III, Medical University of Vienna, Vienna, Austria; 2 Institute of Animal Breeding and Genetics, University of Veterinary Medicine Vienna, Vienna, Austria; 3 Institute of Pathology, Neuropathology and Molecular Pathology, Medical University of Innsbruck, Innsbruck, Austria; 4 Diagnostic & Research Center for Molecular Biomedicine, Institute of Pathology, Medical University of Graz, Graz, Austria; University of Cordoba, SPAIN

## Abstract

The aim of this study was to investigate whether the lack of signal transducer and activator of transcription 5 (STAT5) in mature adipocytes of obese mice (*Stat5*^*Adipoq*^ mice) improves glucose and lipid metabolism as previously observed in lean mice. Male *Stat5*^*Adipoq*^ mice and their wild type (WT) littermates were fed high-fat diet (HFD). Effects of adipocyte STAT5 deficiency on adiposity as well as on glucose and lipid metabolism were determined under *ad libitum* feeding and after weight loss induced by calorie restriction. Compared to WT mice, obese *Stat5*^*Adipoq*^ mice showed modestly accelerated weight gain and blunted depletion of fat stores under calorie restriction (reduction in % body fat after 3 weeks: WT, -9.3±1.1, *vs Stat5*^*Adipoq*^, -5.9±0.8, p = 0.04). No differences were observed between S*tat5*^*Adipoq*^ and WT mice with regard to parameters of glucose and lipid metabolism including basal glycaemia, glucose tolerance, and plasma triglycerides. In conclusion, STAT5 deficiency in the adipocyte of HFD-fed obese mice was associated with increased fat accumulation. In contrast to previous findings in lean mice, however, lipid accumulation was not associated with any improvement in glucose and lipid metabolism. Our results do not support adipocyte STAT5 as a promising target for the treatment of obesity-associated metabolic derangements.

## Introduction

The balance between the storage and mobilisation of lipids in adipose tissue plays a central role in the development of obesity and its associated disturbances, which include the metabolic syndrome, fatty liver disease, and type 2 diabetes. In this context, an increased capacity to store lipids in subcutaneous white adipose tissue (WAT) seems to protect from the metabolic consequences of obesity [1]. Clinical utility of this mechanism is exemplified by thiazolidinediones, whose anti-diabetic effect seems to be mediated by the promotion of lipid accumulation in white adipose tissue. By trapping lipids in the adipocyte, thiazolidinediones are believed to protect from accumulation of ectopic fat as well as of insulin desensitising lipid intermediates in glucoregulatory tissues such as liver, skeletal muscle, and pancreas [2–5]. Drugs that enhance the lipid storage capacity of WAT therefore represent a therapeutic option for the metabolic derangements in selected patients with obesity.

The signal transducer and activator of transcription (STAT5) plays a central role in adipose tissue with involvement in the regulation of adipogenesis [6], adipocyte differentiation [7], fat browning [8,9], adaptations to food restriction [10], brown adipose tissue thermogenesis [10], and lipolysis [11]. Specifically, there is evidence that STAT5A and STAT5B mediate the mobilisation of lipid from the adipocyte induced by growth hormone (GH) [12,13], albeit recent findings suggest that GH may modulate lipolysis also in a STAT5-independent manner [14]. We recently reported that male mice lacking both STAT5 proteins in their mature adipocytes (*Stat5*^*Adipoq*^ mice) show impaired lipolysis in white adipose tissue and increased accumulation of subcutaneous fat mass [11]. Remarkably, the impaired lipolysis observed in lean *Stat5*^*Adipoq*^ mice was associated with improved whole-body insulin sensitivity [11]. While these attributes may in part be sex-specific [14], similar effects have been reported in mouse models with adipocyte-specific deletion of adipose triglyceride lipase (ATGL) [15] as well as of other mediators of GH-signalling (i.e., GH receptor [16], JAK2 tyrosine kinase [17]).

As of yet, the evidence for altered lipid trafficking and metabolic improvement due to adipocyte STAT5 deficiency stems from chow-fed lean *Stat5*^*Adipoq*^ mice [11,14,18]. In the present study, we aimed at investigating whether STAT5 deficiency exerts positive metabolic effects also in mice challenged with a high-fat diet (HFD, i.e. under conditions of obesity, insulin resistance, and lipid overconsumption). If so, adipocyte STAT5 could become a candidate target for therapeutic intervention in selected patients with obesity. We assessed several parameters of glucose and lipid metabolism in obese *Stat5*^*Adipoq*^ mice under HFD and under weight loss induced by food restriction. In contrast to previous findings in conventionally fed lean *Stat5*^*Adipoq*^ mice [11,18], we show that metabolic improvement is absent in the obese state.

## Methods

### Animals and treatment

As previously described in detail, mice with an adipocyte-specific deficiency in STAT5 (*Stat5*^*Adipoq*^) were generated by crossing Adipoq-Cre with *Stat5a/b* floxed mice on a C57BL/6J background [11]. Adipoq-Cre is specifically active in all white and brown mature adipocytes, independent of the location of the fat depot. Accordingly, STAT5 deficiency has previously been documented in epigonadal and in subcutaneous adipose tissue from *Stat5*^*Adipoq*^ mice [11,19].

**Fig 1:**
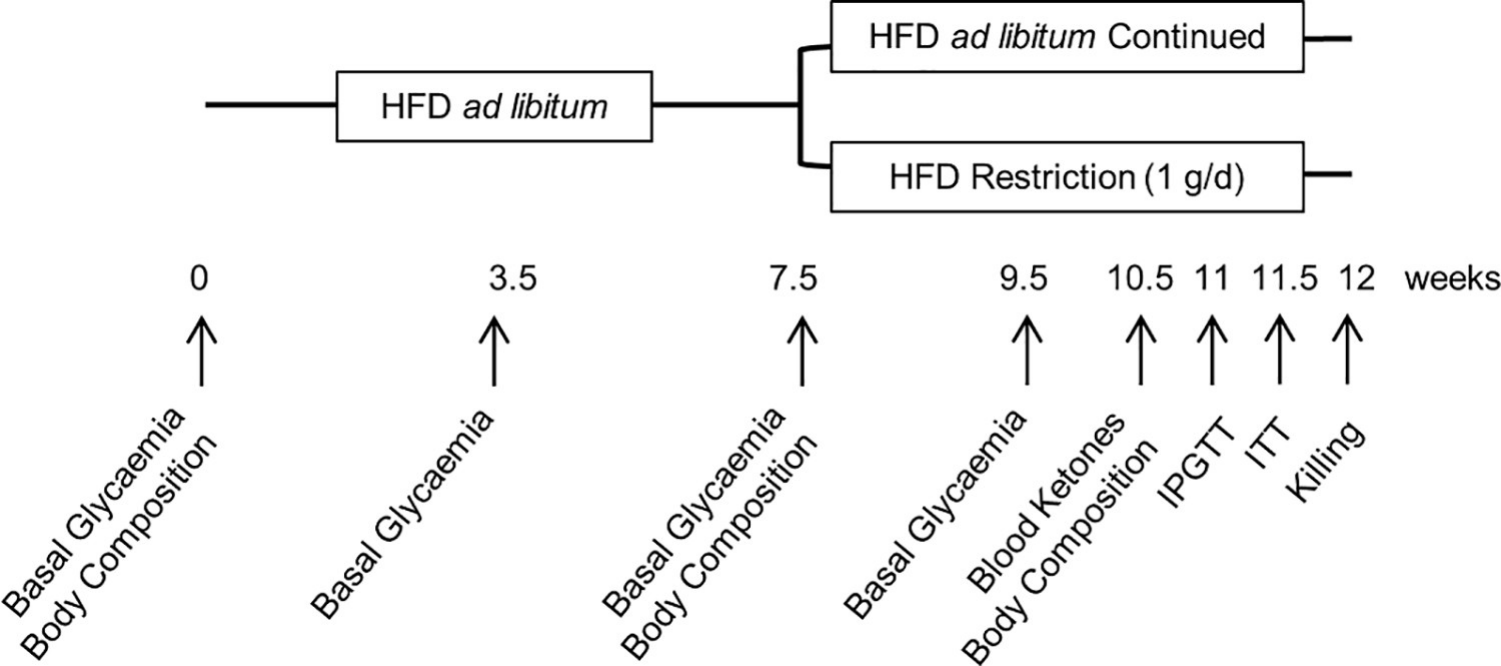
Schematic representation of the study protocol. Feeding of high-fat diet (HFD) was initiated in mice lacking STAT5 in their adipocytes (*Stat5*^*Adipoq*^) and in their wild type controls (WT) when they were approximately 13 weeks old; the HFD was continued for the following 7.5 weeks. Thereafter, mice of both genotypes were divided into two groups each, one fed HFD *ad libitum*, one fed HFD restrictedly (1 g/d) for another 4.5 weeks. Repeated measurements and tests were made at the indicated time points. IPGTT–intraperitoneal glucose tolerance test; ITT–insulin tolerance test.

In the present study, male *Stat5*^*Adipoq*^ mice and their corresponding Cre-negative controls (wild type, WT) were housed at room temperature under an artificial 12 h dark/12 h light cycle. They had free access to tap water and conventional carbohydrate-rich chow diet (sniff R/M-H; sniff Spezialdiäten GmbH; Soest, Germany) until an age of approximately 12–13 weeks. As depicted schematically in Fig 1, animals were then switched to HFD (60% of calories as fat; diet D12492 from Research Diets Inc., New Brunswick, NJ, USA), which is known to cause obesity, insulin resistance, and glucose intolerance in C57BL/6 mice [20]. After 7.5 weeks with free access to HFD, mice of both genotypes were divided into two groups each, with groups of the same genotype matched for weight, glycaemia, and body composition. One group of each genotype continued on HFD *ad libitum*. The other groups were subjected to calorie restriction (daily portions of 1 g HFD per mouse) in order to investigate whether the impairment of lipolysis by STAT5 deficiency dampens the recruitment of lipid stores. Twelve weeks after starting the HFD feeding, mice were deeply anaesthetised with isoflurane and killed by bleeding via heart puncture. Blood and tissues were collected and stored frozen or fixed in formaldehyde solution for subsequent analysis.

All experiments and applied procedures were approved by the Austrian Federal Ministry of Science and Research (approval # GZ: 66009/0159-II/3b/2013).

### Experimental readouts

Body weight was documented approximately twice weekly. The time schedule of further measurements and metabolic tests is graphically summarised in Fig 1. Before starting HFD feeding (0 weeks) as well as 7.5 and 9.5 weeks thereafter, we measured basal blood glucose (8 h fasted; StatStrip Xpress Glucose Meter, Nova Biomedical, Data Sciences International, St.Paul, MN, USA) and body composition (lean and fat mass; Body Composition Analyser, EchoMRI, Houston, TX, USA). Blood ketones were measured at 10.5 weeks (FreeStyle Precision Neo, Abbott, Chicago, IL, USA). During the last week of the experimental protocol, an intraperitoneal glucose tolerance test (IPGTT) and an insulin tolerance test (ITT) were performed. For the IPGTT mice were fasted for 8 h, before the tip of the tail was pricked and basal blood glucose was measured. Immediately thereafter, the IPGTT started with an intraperitoneal injection of 33% (wt/vol) glucose solution (1.5 g/kg body weight in a volume of 4.5 μl/g body weight). Further measurements of blood glucose followed at 30, 60, 90, 120, and 150 min after glucose injection. For the ITT, mice were fasted for 4 h and, after measuring basal glycaemia, 0.75 U/kg human insulin (Sigma Aldrich, St.Louis, MO, USA) was injected intraperitoneally (5 μl/g body weight, diluted in saline). Blood glucose was determined 15, 30, 45, and 60 min thereafter. In order to avoid severe hypoglycaemia in the food restricted groups, their ITT was discontinued at 30 min by intraperitoneal injection of glucose (2 g/kg body weight). Terminal plasma and tissue samples were harvested under deep anaesthesia and immediately after killing the animals. Plasma insulin (Ultrasensitive Mouse Insulin ELISA, Mercodia, Uppsala, Sweden), free fatty acids (NEFA-HR-2 Assay, Wako Chemicals GmbH, Neuss, Germany), triglycerides (Triglyceride Determination Kit, Sigma Aldrich, St.Louis, MO, USA), aspartate aminotransferase and alanine aminotransferase (using a test-slide based blood chemistry analyser; IDEXX VetTest 8008, IDEXX Laboratories, Westbrook, OK, USA) were determined subsequently. Hepatic steatosis was determined after triglyceride extraction according to Folch *et al*. [21], as well as by histological scoring. To investigate potential reactive compensation of STAT5 deficiency in adipocytes through activation of the signal transducer and activator of transcription 3 (STAT3) pathway, as it is known to occur in STAT5-deficient liver [22,23], we measured the expression and activation (i.e., tyrosine phosphorylation) of STAT3 in epidydimal fat with Western blot analysis. Browning of white adipose tissue was also examined by Western blot measurement of the expression of uncoupling protein-1 (UCP1) in inguinal adipose tissue, a fat depot with a high propensity to browning [24].

### Histology

Immediately after killing, specimens of liver were fixed in 4% neutral buffered formaldehyde solution. They were embedded in paraffin and dewaxed 4 μm thick sections were stained with haematoxylin and eosin (H&E) for microscopic examination for degree of steatosis (scored in steps of 10 from 0 = no steatosis, to 100 = maximal steatosis), as well as for the predominant location of steatosis according to Rappaport zones. Examination and scoring were conducted by a board certified pathologist (J.H.) in a blinded study setup.

### Western blot

Samples of epidydimal fat were homogenized in 40 mM ß-glycerophosphate, 2 mM sodium orthovanadate, 20 mM MOPS, 30 mM sodium fluoride, 10 mM sodium pyrophosphate, 2 mM EGTA, 5 mM EDTA, pH 7.4 and complete™ Protease Inhibitor Cocktail (Roche, Nutley, NJ, USA). After centrifugation of the lysates (13,000 g, 20 min, -3°C) protein concentrations were measured with the Pierce BCA Protein Assay Kit (ThermoFisher Scientific Inc., Waltham, MA, USA). Protein extracts were separated on 4–12% NuPAGE gels (Invitrogen, Carlsbad, CA, USA) and blotted onto PVDF-FL membranes for fluorescence detection (Immobilon, Merck-Millipore, Burlington, MA, USA). Membranes were blocked at room temperature for 1 h in Odyssey LI-COR Blocking Buffer (LI-COR Biosciences, Lincoln, NE, USA) diluted 1:1 in TBS and were incubated overnight at 4°C with primary antibodies (Ab).

For the detection of STAT3 and phospho-STAT3 Rabbit primary Ab against STAT3 and phospho-STAT3 Tyr705 (#4904 and #9145, Cell Signaling Technology, Danvers, MA, USA) were used at a dilution of 1:1,000 in TBST, and Mouse primary Ab against β-actin as house-keeping protein (#ab8226, Abcam, Cambridge, UK) were used at a dilution of 1:5,000 in TBST. For the detection of UCP1 we used Rabbit primary Ab against UCP1 (#ab23841, Abcam) at a dilution of 1:500 in TBST and Mouse primary Ab against β-actin (C4) as house-keeping protein (sc-4778, *Santa Cruz* Biotechnology, Inc., Dallas, Texas, USA) at a dilution of 1:250 in TBST.

After being washed three times with TBST, the membranes were incubated for 1 h at room temperature with secondary Ab against Mouse and Rabbit IgG (P/N 926–68070 and P/N 926–32211; LI-COR Biosciences) diluted 1:10,000 in Li-Cor Blocking Buffer. Membranes were washed three times in TBST and signal was detected using the LI-COR Odyssey imaging system (LI-COR Biosciences) and quantified with LI-COR ImageStudio (Version 5.2).

#### Statistical procedures

Results are given as means±SEM. p-Values were calculated in the context of an exploratory data analysis using two-tailed unpaired t-test (except for plasma insulin, which was not normally distributed and therefore analysed by Mann-Whitney rank test). A p<0.05 was considered significant.

## Results

### Effects of Stat5^Adipoq^ genotype on weight and fat mass

**Fig 2:**
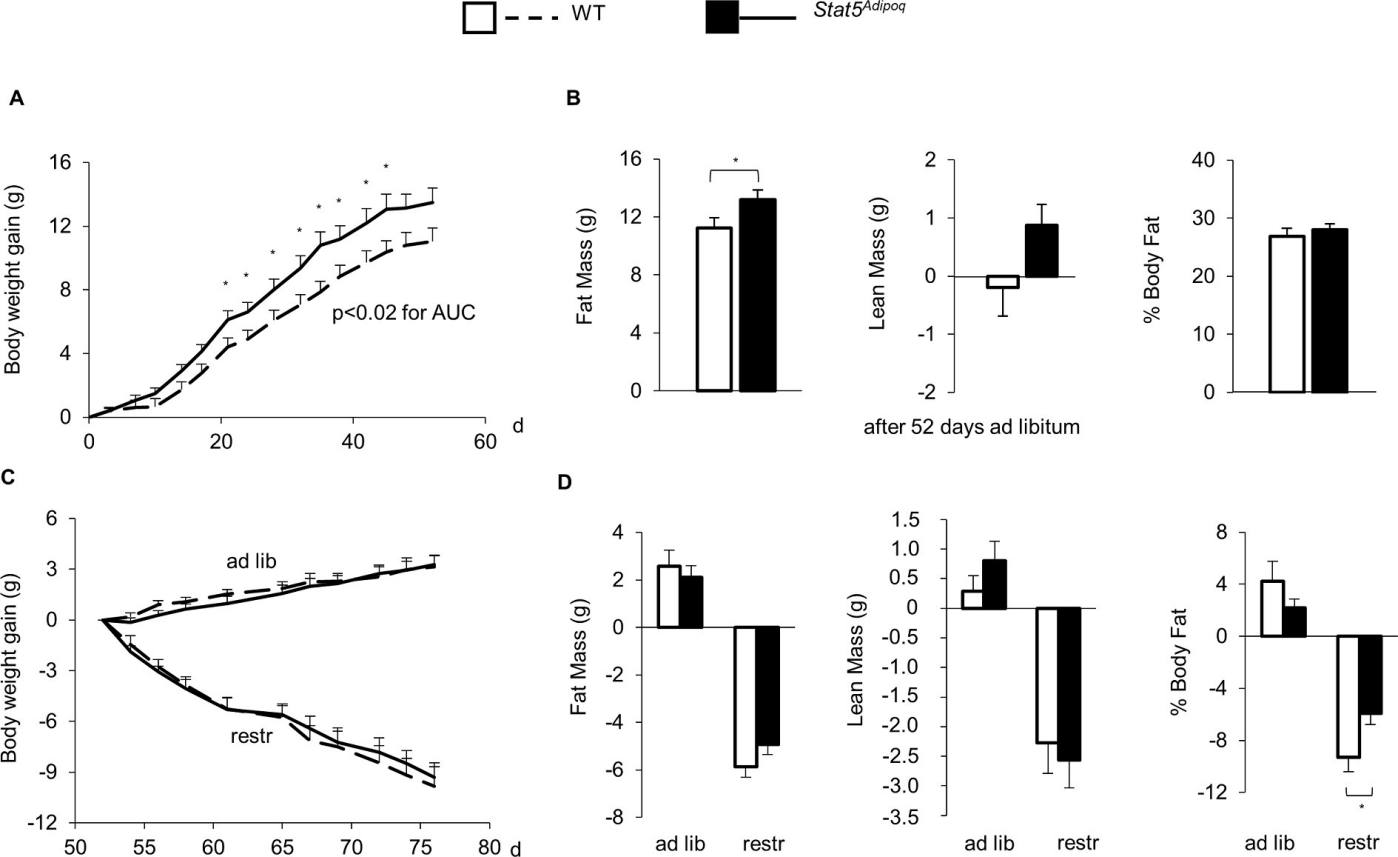
Effects of STAT5 adipocyte deficiency on body weight and body composition. Weight gain (A) and changes in body composition (B) of male mice lacking STAT5 in their adipocytes (*Stat5*^*Adipoq*^; n = 20) and their wild type controls (WT; n = 16) fed high-fat diet *ad libitum* for 7.5 weeks. Thereafter, mice of both genotypes were divided into two groups each, one fed HFD ad libitum (ad lib), one fed HFD restrictedly (1 g/d) (restr). Effects on weight gain (C) and body composition after 3 weeks (D) are shown. Means±SEM; * p<0.05 WT *vs Stat5*^*Adipoq*^.

When HFD feeding started at an age of 12–13 weeks, WT and *Stat5*^*Adipoq*^ mice had similar body weight (WT, 26.7±0.9, *vs Stat5*^*Adipoq*^, 27.4±0.7 g), and body composition (% fat mass: WT, 12.4±1.4, *vs Stat5*^*Adipoq*^, 13.4±1.0%). When the consumption of calories and lipid was stimulated by free access to HFD over the subsequent 7.5 weeks, *Stat5*^*Adipoq*^ mice showed increased weight gain (Fig 2A) with a final mean absolute body weight 8.5% higher than WT mice (WT, 37.7±1.1, vs *Stat5*^*Adipoq*^, 40.9±1.3 g; p = 0.077). Moreover, *Stat5*^*Adipoq*^ mice accumulated more fat mass compared to WT mice (Fig 2B; p = 0.0495).

We then applied a food restriction protocol to HFD fed mice in order to assess whether impaired lipolysis ascribed to STAT5 deficiency [11] affects the recruitment of stored fat and decelerates loss of body fat and weight. Food restriction caused a significant weight loss of similar extent in *Stat5*^*Adipoq*^ and in WT mice (Fig 2C). However, over the 3 weeks of restricted diet, the S*tat5*^*Adipoq*^ mice lost significantly less % of body fat than WT mice (Fig 2D, WT, -9.3±1.1 vs *Stat5*^*Adipoq*^, -5.9±0.8%, p = 0.035).

### Effects of Stat5^Adipoq^ genotype on glucose and lipid metabolism

**Fig 3:**
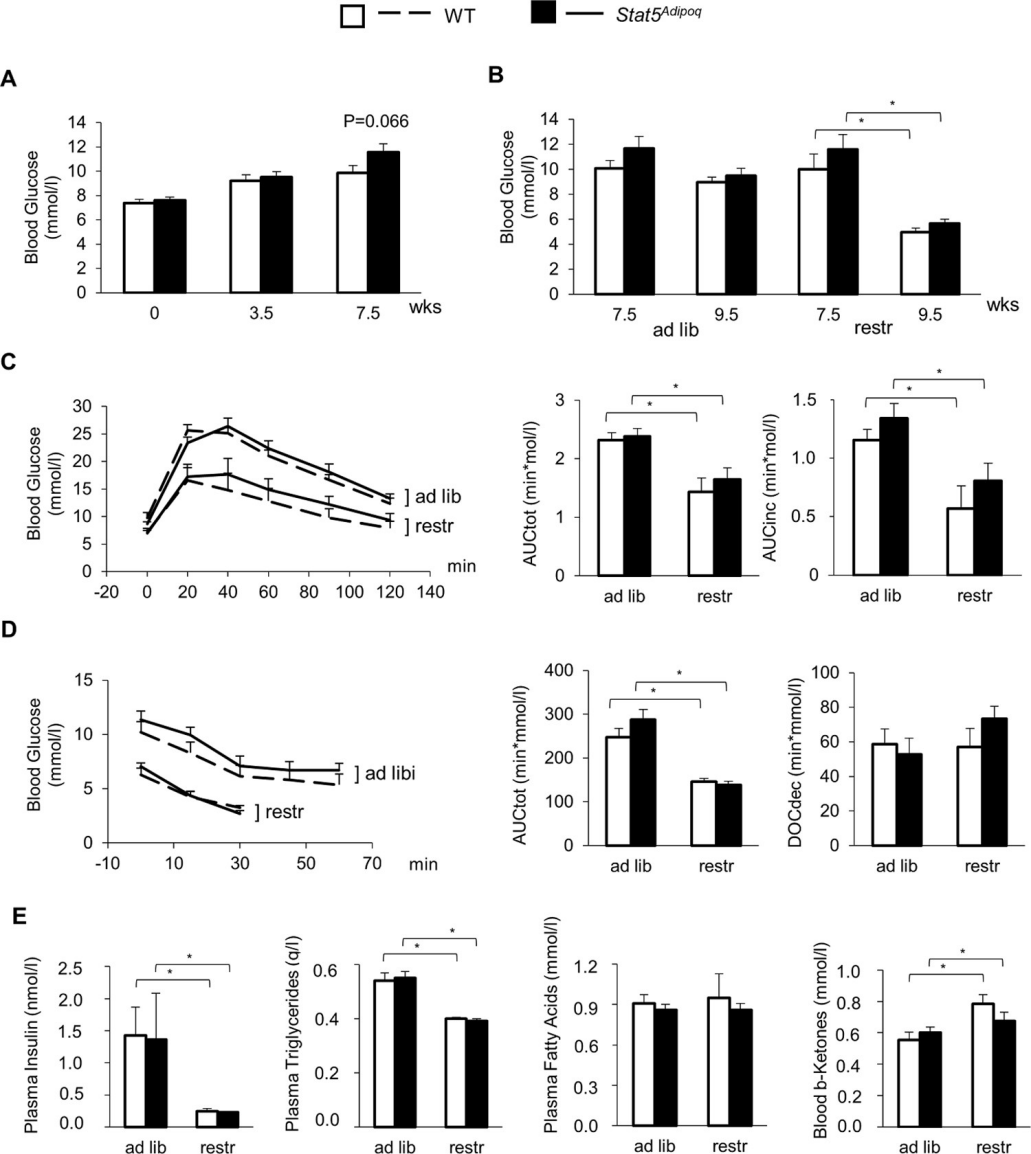
Effects of STAT5 adipocyte deficiency on glucose and lipid metabolism. Basal blood glucose of male mice lacking STAT5 in their adipocytes (Stat5^Adipoq^; n = 20) and their wild type controls (WT; n = 16) fed high-fat diet (HFD) *ad libitum* for 7.5 weeks (A). Thereafter, mice of both genotypes were divided into two groups each, one fed HFD *ad libitum* (ad lib), one fed HFD restrictedly (1 g/d) (restr). After 2–3 weeks, basal blood glucose (B), glucose tolerance test (C), insulin tolerance test (AUC and DOC refer to 0–30 min) (D), and circulating insulin, triglycerides, free fatty acids, and β-ketones (E) were assessed. Means±SEM; n = 7–12 per group; AUCtot, total area under the curve; AUCinc, incremental area under the curve; DOCdec, decremental area over the curve; *p<0.05 ad lib vs restr; no significant differences for WT vs Stat5^Adipoq^.

At the beginning of HFD feeding, WT and *Stat5*^*Adipoq*^ showed no significant differences in blood glucose (Fig 3A). At variance to previous evidence for improved glucose homeostasis in lean *Stat5*^*Adipoq*^ mice, we observed a trend towards increased glycaemia after 7.5 weeks of HFD feeding (Fig 3A; WT, 9.9±0.6, *vs Stat5*^*Adipoq*^, 11.6±0.7 mmol/l; p = 0.066). After 2–3 weeks of food restriction and weight loss, both WT and *Stat5*^*Adipoq*^ mice had significantly lower basal glycaemia (Fig 3B, p<0.001 each) and glucose excursions in the IPGTT (Fig 3C, AUC: p = 0.009 each) as well as in the ITT (Fig 3D, AUC: p<0.001 each) compared to the respective *ad libitum* fed controls. However, there was no significant effect of the genotype on any of these parameters (Fig 3B–3D). Concentrations of insulin and triglycerides in plasma decreased under food restriction in *Stat5*^*Adipoq*^ as well as in WT mice (Fig 3E), whereas circulating free fatty acids were unaffected and β-ketones modestly increased in WT mice only. Also for these parameters no effect of *Stat5*^*Adipoq*^ could be found (Fig 3E).

**Fig 4:**
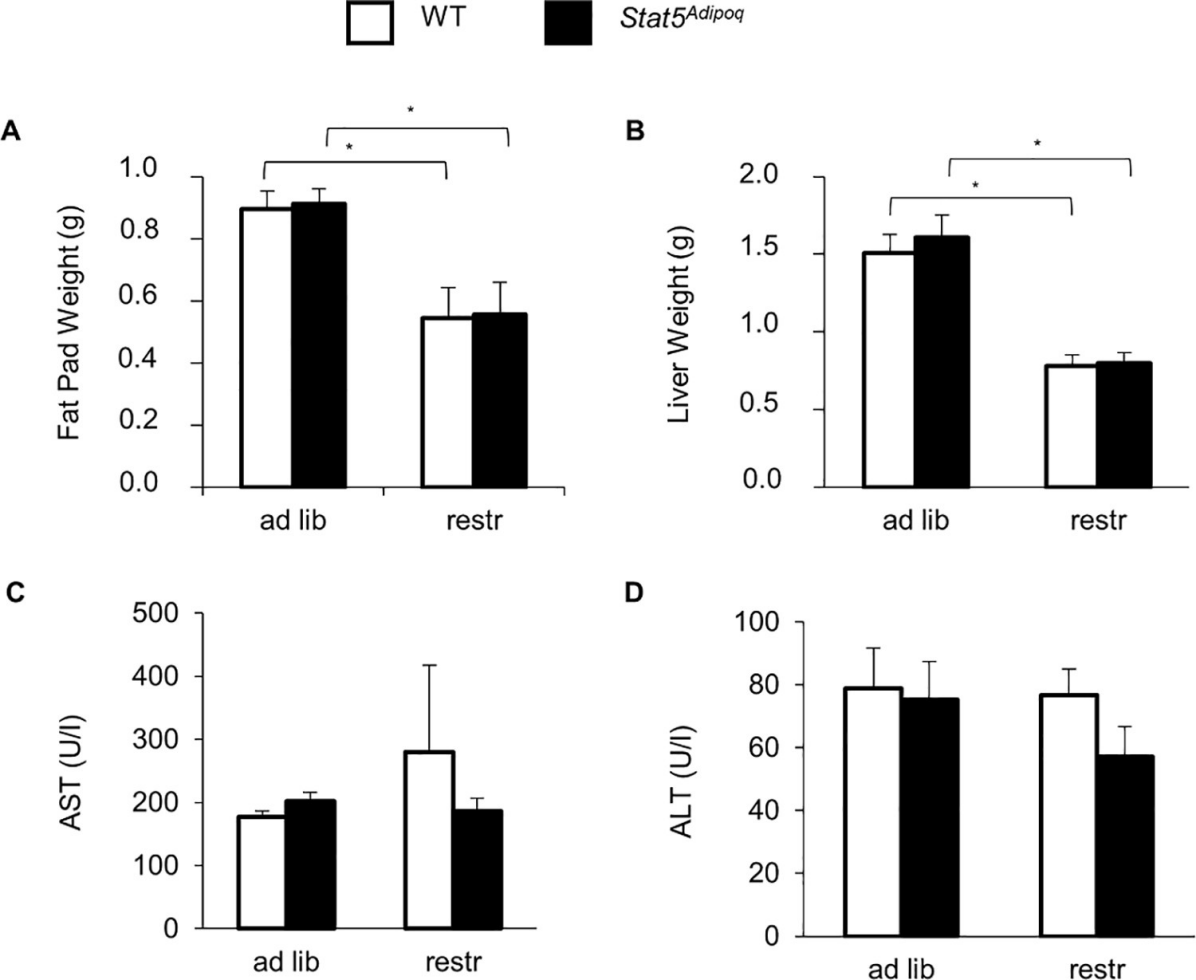
Effects of STAT5 adipocyte deficiency on epidydimal fat and liver. After 7.5 weeks of high fed diet (HFD), 23–24 week-old male mice lacking STAT5 in their adipocytes (*Stat5*^*Adipoq*^) and their wild type controls (WT) were split into two groups, one fed HFD *ad libitum* (ad lib), one fed restrictedly (1 g/d) (restr). After 2–3 weeks, weights of epidydimal fat pad (A) and liver (B), and circulating aspartate aminotransferase (AST; C) and alanine aminotransferase (ALT; D) were assessed. Means±SEM; n = 7–12 per group; *p<0.05 ad lib *vs* restr; no significant differences for WT *vs Stat5*^*Adipoq*^.

**Fig 5:**
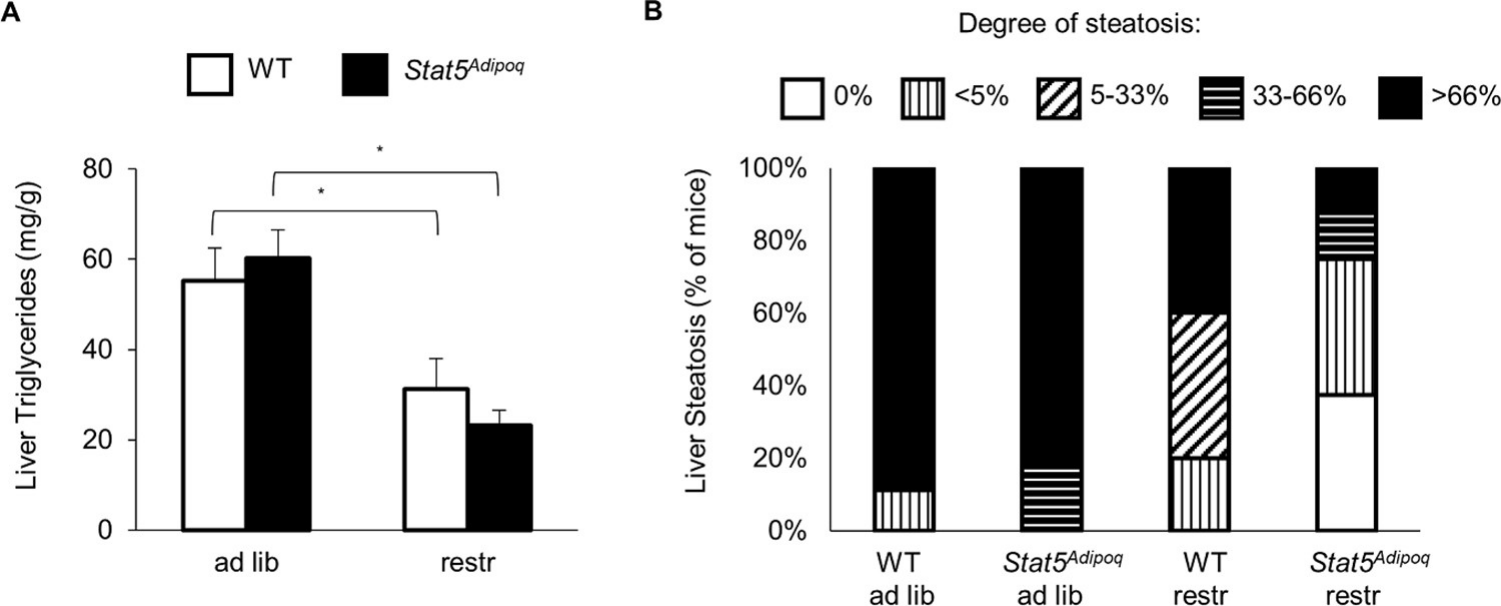
Effects of STAT5 adipocyte deficiency on liver histology. After 7.5 weeks of high fed diet (HFD), 23–24 week-old male mice lacking STAT5 in their adipocytes (*Stat5*^*Adipoq*^) and their wild type controls (WT) were split into two groups, one fed HFD *ad libitum* (ad lib) and one fed restrictedly (1 g/d) (restr) for the following 4.5 weeks. Hepatic steatosis was determined by measuring triglyceride concentration in liver extracts (means±SEM; *p<0.05 ad lib *vs* restr; no significant differences for WT *vs Stat5*^*Adipoq*^; A), as well as by histological score (B). n = 7–12 per group.

Weight loss was also accompanied by reductions in epidydimal fat pad and in liver weights with no differences linked to STAT5 deficiency (Fig 4A and 4B). Aspartate aminotransferase and alanine aminotransferase were similar in all examined mouse groups (Fig 4C and 4D). Consistently, chemical and histological analyses showed a comparable extent hepatic steatosis in the two genotypes (Fig 5).

**Fig 6:**
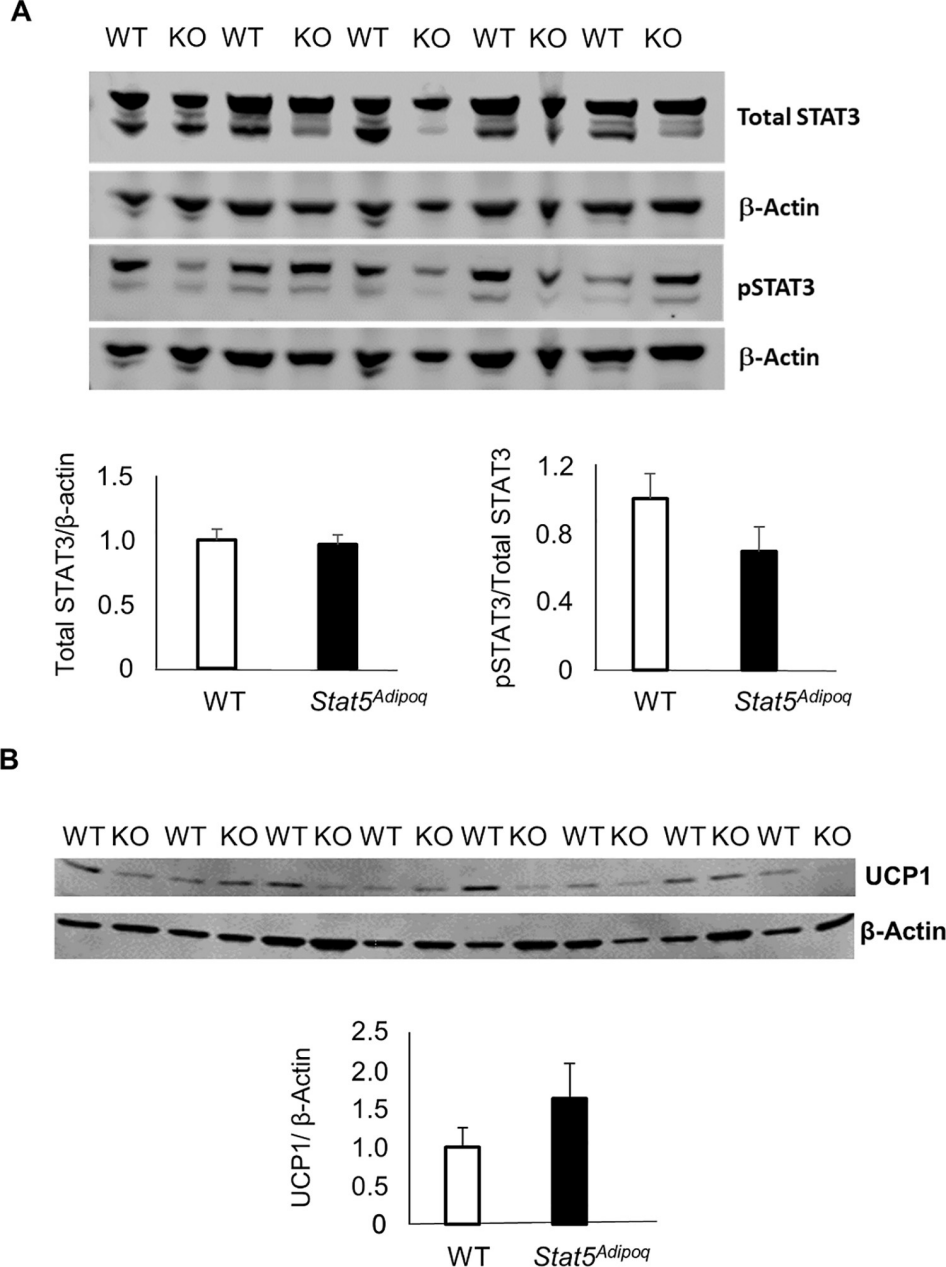
STAT3 expression and phosphorylation in epidydimal fat and UCP1 expression in inguinal fat of mice with adipocyte STAT5 deficiency. Abundance of total STAT3 and of phosphorylated STAT3 (pSTAT3) normalised to β-actin in epidydimal fat (A) and UCP1 expression normalised to β-actin in the inguinal fat (B) from 23–24 weeks old male mice fed high-fat diet *ad libitum* which lack STAT5 in their adipocytes (*Stat5*^*Adipoq*^*/*KO) as compared to their wild type controls (WT). No significant differences WT *vs Stat5*^*Adipoq*^.

The lack of STAT5 signalling affected neither the total expression nor the tyrosine phosphorylation of STAT3 in the epidydimal fat. Likewise, *Stat5*^*Adipoq*^ and WT mice showed no differences in UCP1 expression in the inguinal adipose tissue (Fig 6).

## Discussion

The results of the present study are in line with previous findings that the lack of STAT5 in mature adipocytes of mice can trigger detectable changes in accumulation of fat mass. However, the effect in the obese mice fed HFD was clearly less pronounced than previously found in lean mice [11]. Another study likewise found clear-cut effects on fat accumulation in lean mice lacking STAT5 in adipocytes, but completely failed to detect any difference under HFD [14]. Our obese *Stat5*^*Adipoq*^ mice lost less relative body fat than their WT littermates during food restriction, which suggested impaired lipid recruitment from adipose tissue. Also in this case, the effect was not as marked as previously observed in lean *Stat5*^*Adipoq*^ mice under acute stimulation of lipid mobilisation by starvation (white adipose tissue) [11] or under cold exposure (brown adipose tissue) [11,18].

In lean *Stat5*^*Adipoq*^ mice on carbohydrate-rich food, lipid accumulation in adipocytes was accompanied by improved blood glucose homeostasis and reduced plasma lipids [11]. In contrast, comparable benefits were absent in obese *Stat5*^*Adipoq*^ mice under oversupply of alimentary lipids. Obesity-associated liver damage was also similar in the two genotypes. Of note, the absence of metabolic improvement seen in obese *Stat5*^*Adipoq*^ *versus* wild type mice fed HFD persisted under stringent food restriction and markedly reduced body weight, which implicates that the high fat content of the diet rather than adiposity or calorie overconsumption accounted for the lack of metabolic benefit.

One plausible explanation for adipocyte STAT5 deficiency having less metabolic consequences in obese *versus* lean mice would be that release and circulating concentrations of the STAT5 activator GH are markedly reduced in obesity [25–27]. If so, the disruption of minor residual STAT5 activity in the obese could have little impact, whereas blocking the robust GH signal typically prevailing in lean animals could account for the metabolic consequences described previously [11]. Another mechanism putatively contributing to the improvement of metabolic traits in chow fed *Stat5*^*Adipoq*^ mice is the trapping of lipids in adipose tissue, which protects other tissues from accumulation of insulin desensitising lipid metabolites. Under conditions of very high fat intake, however, the capacity of STAT5 deficient adipocytes for lipid trapping could simply not suffice for effective relief of non-adipose organs from oversupply and insulin desensitisation. Another possible cause for the lack of effects induced by STAT5 deficiency is compensation via alternative pathways, as it has been described via activation of STAT3 in STAT5-deficient liver [22,23]. Although we failed to find a compensatory increase in the expression or activation of STAT3 in adipose tissue, we cannot rule out compensation of the metabolic consequences of STAT5-deficiency in adipocytes of obese animals via other mechanisms. Activation of compensatory mechanisms in other tissues, even through the ubiquitously expressed STAT5 itself, may likewise have occurred.

## Conclusion

The results of the present study confirmed increased lipid storage in the STAT5 deficient adipocyte in mice. Nevertheless, the regulatory impact of STAT5 on adipocyte lipid turnover appears quantitatively not sufficient for counteracting the metabolic consequences of high alimentary lipid consumption and obesity. Hence, our findings moderate speculations about adipose STAT5 as a target for the treatment of obesity associated metabolic disturbances.

## Supporting information

S1 Raw images (PDF)
