# Supplementary material for: Adipocyte STAT5 deficiency does not affect blood glucose homeostasis in obese mice
Source: PLoS One. 2021 Nov 24;16(11):e0260501. doi: 10.1371/journal.pone.0260501 (PMC8612524; doi:10.1371/journal.pone.0260501)
Supplement: S1 Raw images — (PDF) [file pone.0260501.s001.pdf]

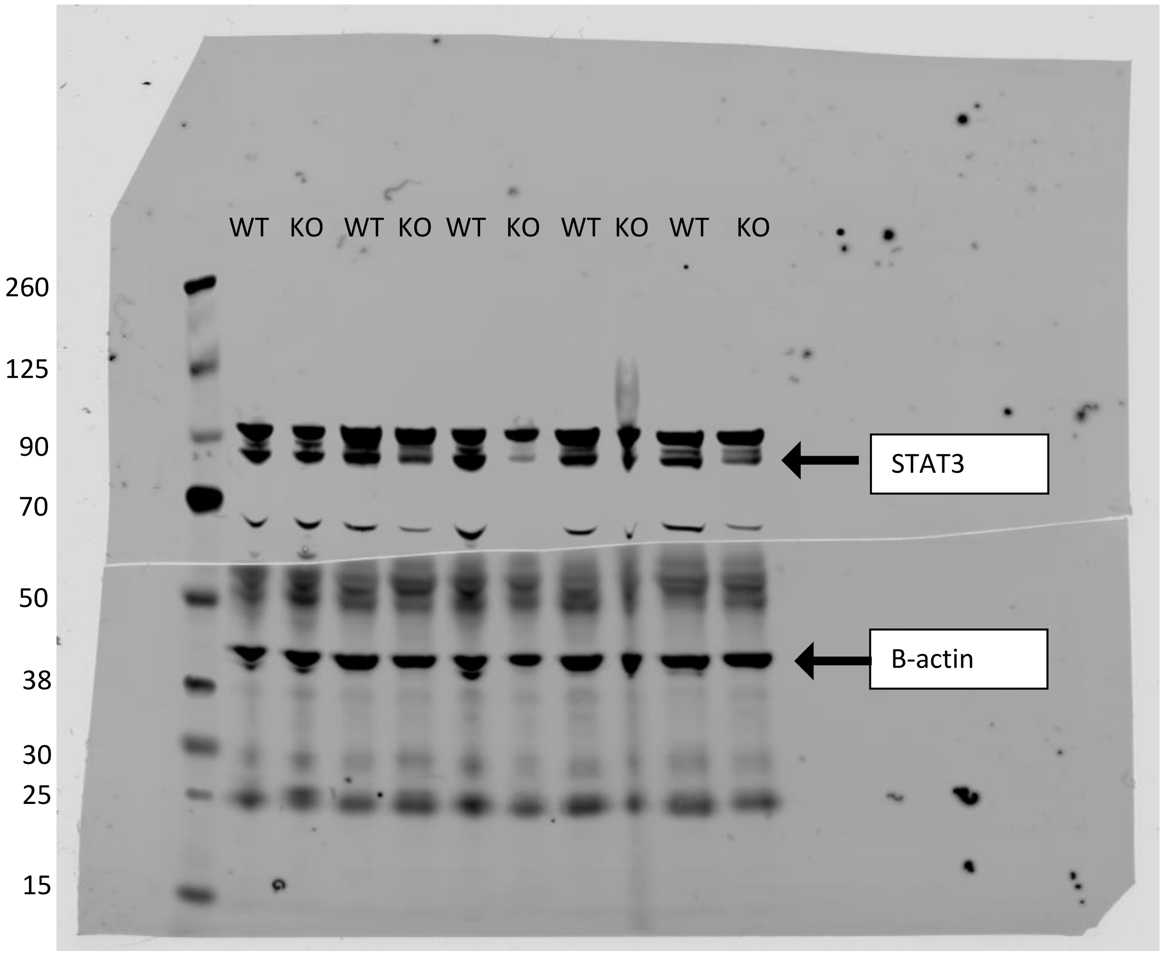

STAT3 Western blot (Fig 6 A)

Signal was detected using the LI-COR Odyssey imaging system (LI-COR Biosciences) and quantified with LI-COR ImageStudio

Marker: Chameleon Duo pre-stained protein ladder

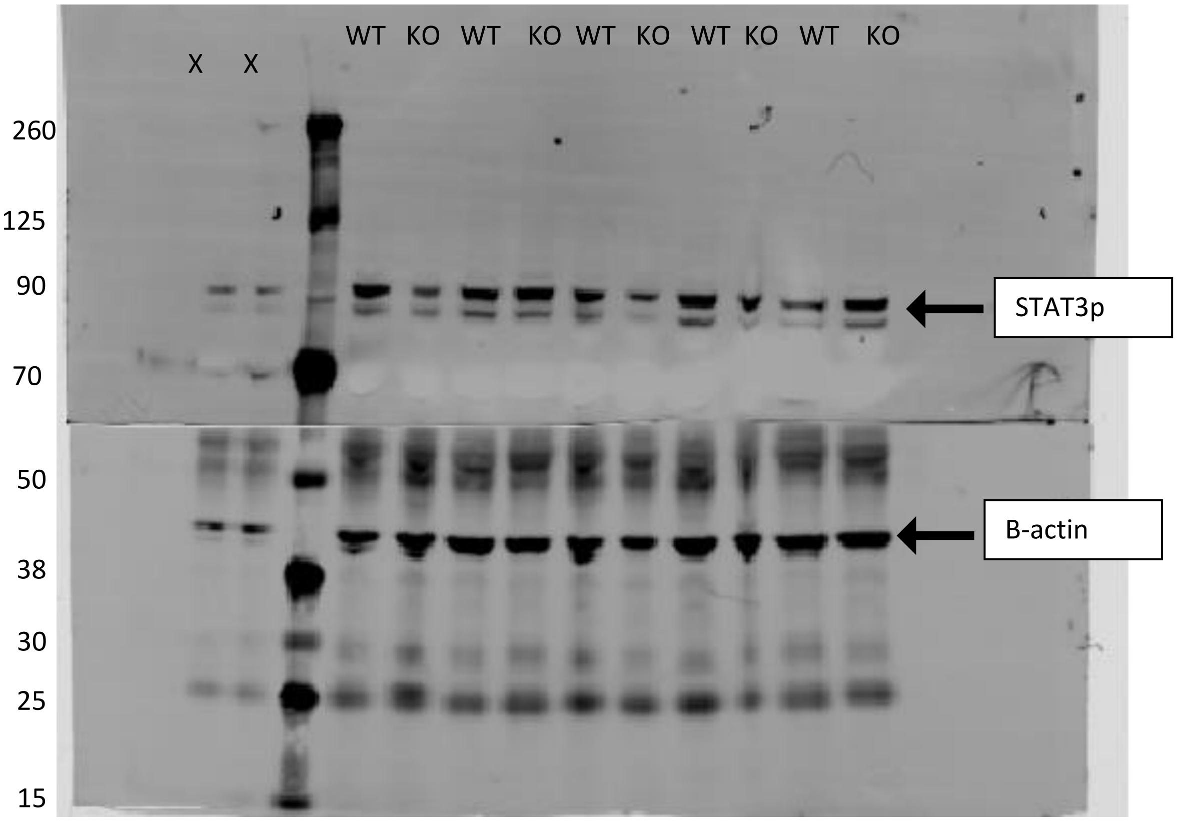

STAT3 phosphorylated Western blot (Fig 6 A)

Signal was detected using the LI-COR Odyssey imaging system (LI-COR Biosciences) and quantified with LI-COR ImageStudio

Marker: Chameleon Duo pre-stained protein ladder

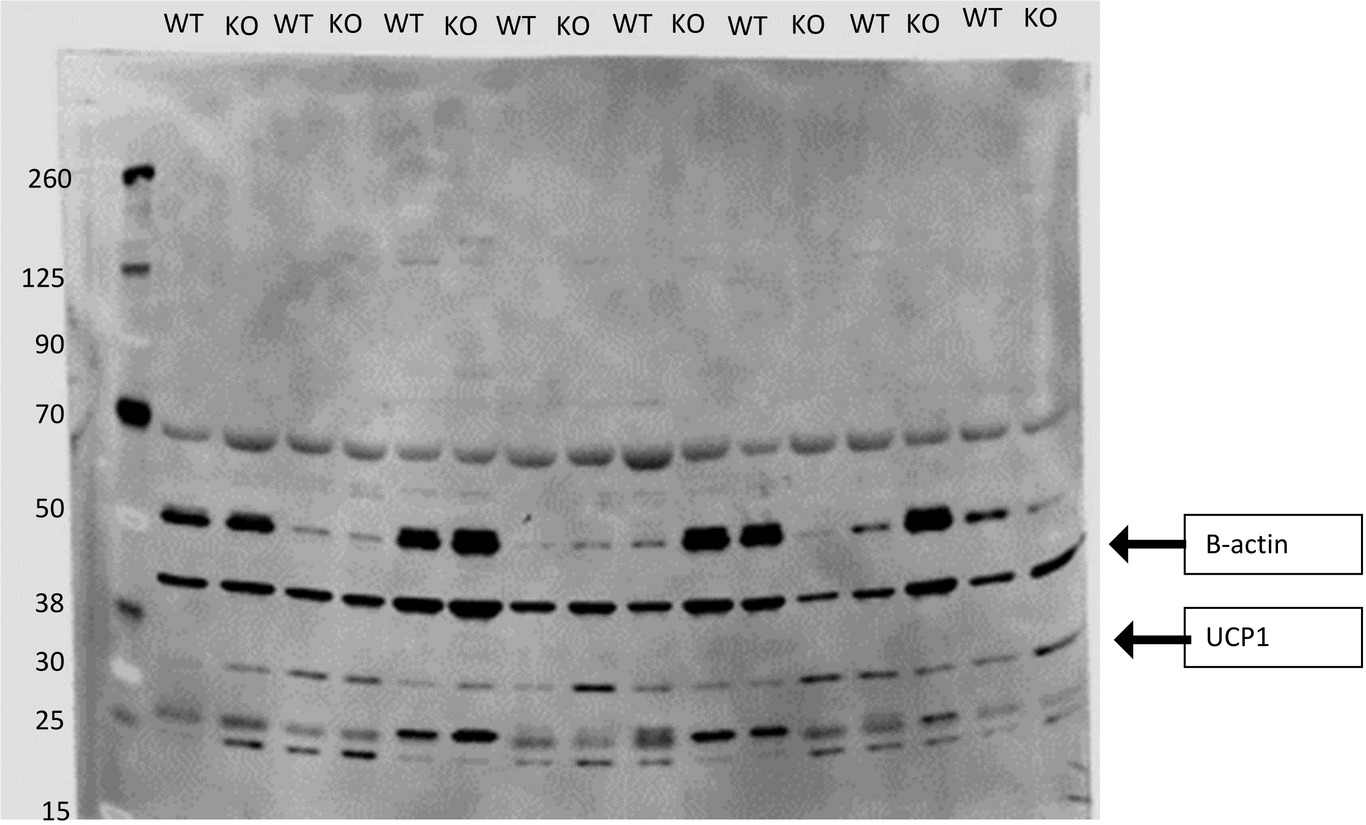

UCP1 Western blot (Fig 6B)

Signal was detected using the LI-COR Odyssey imaging system (LI-COR Biosciences) and quantified with LI-COR ImageStudio

Marker: Chameleon Duo pre-stained protein ladder
